# Pregnancy in a woman undergoing peritoneal dialysis: Management and dialysis options 

**DOI:** 10.5414/CNCS110828

**Published:** 2022-04-29

**Authors:** Rita Veríssimo, Estela Nogueira, João Bernardo, Marta Pereira, Cristina P. Abreu, Noelia Lopez, Cristina Resina, Patrícia Matias, José António Lopes, Patrícia Branco, Luísa Pinto

**Affiliations:** 1 Nephrology Department, Centro Hospitalar Lisboa Ocidental, Hospital de Santa Cruz, Carnaxide, and; 2 Nephrology Department, Centro Hospitalar Lisboa Norte, Lisboa, Portugal; * Equal contribution

**Keywords:** end-stage renal disease, peritoneal dialysis, dialysis dose, pregnancy

## Abstract

Pregnancy in patients with end-stage renal disease on maintenance dialysis is uncommon, with annual incidences reported at 0.3 – 2.7%. Peritoneal dialysis usage in pregnancy has been less reported than hemodialysis, although outcomes are similar. Nowadays, there are insufficient data to establish a generalizable dialysis strategy in pregnant women with end-stage renal disease. As such, decisions should be individualized, depending on clinical factors, residual renal function, and, whenever possible, choice of the patient. We report the case of a 22-year-old patient receiving peritoneal dialysis who delivered a full-term, normal weight, healthy baby with increased dialysis dose achieved by supplementary hemodialysis during pregnancy, thus enabling peritoneal dialysis to be continued until the third trimester and minimizing hemodialysis requirements.

## Background 

Although pregnancy in patients with end-stage renal disease (ESRD) is still a rare event, the incidence of pregnancy in women on hemodialysis (HD) has increased substantially in the last decade, with reported annual incidences at 0.3 – 2.7% [1, 2, 3, 4]. On the other hand, peritoneal dialysis (PD) pregnancy rates are significantly lower and have not changed over the last years [1, 4]. Fertility seems to be lower in PD patients, which has been linked to impairment of normal ovulation by hypertonic dialysate [5]. 

Following conception, infant survival and prematurity do not differ significantly from those of pregnant women on HD, but a systematic review found a higher incidence of small-for-gestational age (SGA) newborns born to women on PD vs. HD (66.7 vs. 31%; p = 0.015) [4]. 

PD offers potential advantages compared to HD, namely preservation of residual renal function (RRF) and continuous daily ultrafiltration, which have been related to improved pregnancy outcomes [6]. However, the lower incidence of pregnancy rates in women on PD and data scarcity have led most authors and guidelines to recommend the switch to HD prior to conception or during the first trimester [1, 4, 7]. 

Nevertheless, long and daily HD schedules are highly demanding for both patient and healthcare facilities. Therefore, in women with significant RRF or those who are unable to immediately switch to intensive HD, maintaining PD during part of the pregnancy or adding intermittent HD could be valuable options and successful cases have been reported [2, 7, 8, 9, 10, 11, 12]. 

The optimal PD prescription in pregnancy remains to be determined and depends on many factors, such as RRF and tolerance to volume dwell, as evidenced in published reports that reveal heterogeneous approaches [9, 10, 11, 12, 13]. 

Herein, we report the case of a successful pregnancy in a PD patient with significant RRF, which was managed with PD until the 25^th^ week and later switched to intensive HD, after a brief period of hybrid dialysis therapy. 

## Case report 

A 22-year-old female from Cape Verde (gravida 1, para 0), with body mass index of 18.3 kg/m^2^ and no personal or family history of kidney disease or other medical conditions was diagnosed with crescentic IgA nephropathy 2.5 years before conception. She progressed to ESRD, and HD was initiated. Later that year, she was transferred to continuous ambulatory PD (CAPD). Her blood pressure was normal. Peritoneal equilibration test (PET) performed 3 months prior to conception, using a 3.86% glucose solution and 4-hour dwell, showed a high dialysate to plasma creatinine ratio (0.83) and a net ultrafiltration volume of 650 mL. At the same time, residual urine output was 2,600 mL per 24 hours, creatinine clearance of 14.9 mL/min/1.73m^2^, and weekly Kt/V 3.5. 

Pregnancy was unplanned and diagnosed at 7 weeks’ gestation. At the time, the patient was being treated with 3 exchanges per day (1.5 L), using 1.36% glucose solutions in 2 exchanges during the day and 1 icodextrin solution overnight. 

Considering her RRF and low urea levels (urea 11 mmol/L) with a rather modest PD dose, the possibility of better preserving RRF and of reducing the risk of SARS-CoV-2 exposure, the multidisciplinary team decided to maintain PD. CAPD prescription was adjusted to 4 and then 5 exchanges per day with 1.8 L of volume dwell. At 11 weeks of gestation, the patient was switched to automated PD (APD) due to abdominal discomfort, urea increase (12.6 mmol/L), and considering she was a high transporter. During the second trimester, she was transferred to PD plus to keep maternal urea levels below 12.5 mmol/L (Table 1). 

As pregnancy progressed and her daily diuresis reduced (diuresis 900 mL per day), consecutive changes were made in PD prescription to increase dialysis adequacy while maintaining patient’s comfort. Volume dwells were reduced, APD time prolonged, cycle numbers increased and CAPD suspended when intra-abdominal pressure reached 16 mmHg. Despite these adjustments, urea levels increased (12.3 mmol/L), and at week 25 the patient was transferred to a hybrid dialysis program. At that point, HD 4 hours thrice a week was added (via a tunneled central venous catheter) to APD, reducing PD hours. However, at week 26, urea level was still high (12.3 mmol/L) and the patient developed exhaustion related to the hybrid technique, with progressive anorexia and signs of malnutrition (albumin 2.5 g/L), despite close follow-up by a nutritionist and dietetic supplements. 

Switching to an intensive HD schedule was then decided (6 hours, 6 times per week, high flux HD, blood flow 400 mL/min, K 3 meq/L, HCO_3_ 28 – 30 meq/L, minimal heparin dose) and the patient improved her nutritional status continuously until the end of gestation. 

Aspirin 150 mg daily was started at 12 weeks, but transiently suspended due to vaginal bleeding at week 12. The pregnancy was further complicated by 1^st^ trimester gestational diabetes controlled with diet and hypothyroidism controlled with levothyroxine. Anemia emerged at week 27 and was treated with darbepoetin (maximum dose 80 µg/week) and IV iron. Vitamin B and D, folic acid, and oral phosphorus supplements were administered. 

Regarding PD complications, at week 22, the patient developed one episode of exit-site infection due to *Corynebacterium tuberculostearicum* and was treated with high-dose amoxicillin (3 – 4 g/day) for 21 days, followed by prophylactic amoxicillin, 500 mg daily, until delivery. 

During pregnancy, serial ultrasound scans were performed at 12, 21, 27, and 34 weeks. In this last scan, the fetus was growing in the 14^th^ percentile, with normal umbilical artery Doppler and normal amniotic fluid volume. 

At 31 weeks gestation, glucocorticoids for fetal maturation were prescribed due to short cervix, and weekly cardiotocographic surveillance was started at 32 weeks. 

Throughout pregnancy, blood pressure control was achieved with non-pharmacological measures until the 36^th^ week, when hypertension developed, although no additional signs of preeclampsia were present (with sflt1/PlGF ratio of 1.9). Nifedipine was initiated (with a maximum dose of 60 mg/day), and the patient was admitted into the hospital for surveillance. 

Preterm premature rupture of membranes occurred at 36 weeks + 5 days, and labor was induced. A normal delivery occurred at 36 weeks + 6 days, and the newborn weighted 2,335 g, with Apgar scores 10/10 at 5/10 minutes, respectively. The baby was immediately breastfed and did not need intensive care admission. 

Mother and child were discharged at the 5^th^ day postpartum. PD was restarted a week after delivery. Two weeks postpartum, the patient had an episode of peritonitis, with no agent identified, successfully treated with cefazolin and ceftazidime. 

Two months after delivery, a PET was performed, showing a residual kidney function of 5 mL/min/1.73m^2^, a weekly Kt/V of 2.45, D/P creatinine ratio of 0.77, a net ultrafiltration of 540 mL with a 4-hour dwell using a 3.86% glucose solution and residual urine output of 1,000 mL per day. 

## Discussion 

Historically, patients on dialysis were discouraged by their physicians to get pregnant as outcomes were very poor [14, 15]. During the last two decades we have assisted to a shift in this paradigm, especially following Hladunewich et al.’s [1] studies that revealed a dramatic improvement in live births (48 vs. 85%), in gestational age at delivery (27 vs. 36 weeks) and in birth weight (1,748 vs. 2,118 g) with intensive HD schedules (36 vs. 20 hours/week) [16, 17, 18]. High serum urea concentrations have been proven to be responsible for many pregnancy complications. Hence, intensifying dialysis dose has been recommended with a target of pre-dialysis urea concentration of less than 12.5 mmol/L [13, 14]. If pregnancy on dialysis is contemplated, some authors suggest increasing the dialysis dose or switch to nocturnal HD to achieve an improvement in the hypothalamic-pituitary-gonadal axis function and raise the possibility of a pregnancy. 

Pregnancy after renal transplantation is still considered the best option for ESRD patients of childbearing age, as it is associated with better maternal and perinatal outcomes. However, for some women, the window of opportunity can be lost due to organ shortage, hyperimmunization, or late pregnancy planning. Thus, pregnancy on dialysis could be the only option, but careful counseling must be carried out, as it is still highly challenging and associated with significant maternal, obstetric, and perinatal complications. Multidisciplinary management by a specialized team, including nephrologist, obstetrician, and neonatologist is of paramount importance to optimize maternal and fetal outcomes in this population [3, 8, 18, 19, 20, 21]. 

ESRD patients have at least a 2-fold risk of adverse outcomes during pregnancy, including miscarriage, placental detachment, premature rupture of membranes, polyhydramnios, preterm birth, aggravation of arterial hypertension, preeclampsia/eclampsia, hemorrhage, and maternal death [1, 22]. 

During pregnancy, PD offers some benefits over HD, namely more continuous and less aggressive daily ultrafiltration, RRF preservation, no need for anticoagulation, and more stable urea levels which could theoretically reduce the risk of polyhydramnios [23]. However, PD is also associated with specific complications, including peritonitis, hemoperitoneum, catheter malposition, catheter-related pain and flow issues, abdominal discomfort due to uterine distension [2, 3, 7, 8, 9, 10, 11]. PD prescription can be challenging particularly in advanced stages of pregnancy, requiring a reduction in dwell volume combined with multiple exchanges over a prolonged period to maintain adequate clearance. Tidal exchanges have been shown to be effective in easing abdominal symptoms. Unsurprisingly, the best individual dialysis technique is yet to be determined. 

Literature shows that in most pregnancies with ESRD HD was used as dialysis modality, especially following the Toronto study [19]. Fertility rates in PD patients seem to be lower than in HD patients, as confirmed by an analysis of the ANZDATA registry, which revealed 2.54 pregnancies per 2,000 patient-years among HD patients vs. 1.06 pregnancies among PD patients [24]. Despite the lower rate, when it occurs, pregnancy on PD has similar outcomes, except for an increased incidence of SGA newborns [1, 2, 9, 10, 11, 20]. The lack of evidence and experience in PD management during pregnancy drive most clinicians to switch to HD at diagnosis or early in the first trimester [1, 13, 25]. 

However, in patients that are unwilling or unable to switch to HD, or in patients with significant RRF, PD could be used to delay intensive HD schedules or as part of a hybrid dialysis approach, where PD is maintained, and intermittent HD is added according to urea levels. Several reports of these alternative strategies have been proven successful and could relieve the burden of daily dialysis for at least part of the pregnancy [1, 2, 3]. 

In our case, the patient’s desire to continue on PD, the significant RRF, and the risk of its risk of RRF loss in early pregnancy, because exposure to SARS-CoV-2 is highest in dialysis facilities, led the authors to pursue PD as long as urea levels and PD tolerance were acceptable. Several adjustments had to be made in PD during pregnancy in order to improve dialysis efficacy and patient’s tolerance as exhibited in Table 1. In our case, hybrid technique could not be maintained for long as the patient felt too tired and signs of malnutrition increased. Once the switch to intensive dialysis took place, the patient recovered her appetite, and her nutrition status improved considerably. It is important to emphasize that PD dose should not be adjusted using Kt/V but instead urea levels and clinical symptoms as recommended by available evidence and guidelines [1, 2, 13, 26, 27]. 

Regarding delivery, it is recommended to induce labor at 37 weeks of gestation allowing a planned delivery, with drained PD dialysate or heparin free HD [1, 10]. Vaginal route is preferred, and PD can be restarted 24 hours after delivery. Increased risk of peritonitis in the post-partum period has been described, which happened in our case. If a cesarean section is needed, 6 weeks of pause is advisable before PD initiation. 

## Conclusion 

Although evidence and experience in the management of PD in pregnant women with ESRD are lacking, this can be a valuable option as a bridge to HD or as part of hybrid dialysis therapy in order to preserve RRF and reduce the burden of intensive dialysis schedules. Dialysis strategies should be tailored whenever possible according to the patient’s individual clinical and personal context. This case highlights the clinical challenges in pregnant PD patients and the importance of collaboration between healthcare facilities that reunite multidisciplinary teams specialized in nephro-obstetric care. 

## Funding 

None to declare. 

## Conflict of interest 

None declared. 

**Table 1.** Urea levels and dialysis prescription evolution during pregnancy.

Gestational age	Urea mmol/L	Dialysis prescription
Week 8	11	• CAPD 4 exchanges (icodextrin + 1.36% glucose solutions)
		◦ 1,500 mL per exchange
Week 10	7.3	• CAPD 5 exchanges (icodextrin + 1.36% glucose solutions)
		◦ 1,800 mL per exchange
Week 11	8.0	• APD 8 h 4 cycles (1.36% glucose solutions)
		◦ 1,900 mL per exchange
		• Icodextrin 1,300 mL during the day
Week 15	8.2	No change
Week 20	9.7	• APD 8 h 4 exchanges (1.36% glucose solutions)
		◦ 1,800 mL per exchange
		◦ Icodextrin 1,300 mL during the day
		• CAPD 2 exchanges (2 × 1.36% glucose solutions)
		◦ 1,500 mL per exchange
Week 21	12.7	• APD 10 h 6 exchanges (1.36% glucose solutions)
		◦ 1,700 mL per exchange
Week 22	10.5	• APD 10 h 7 exchanges (1.36% glucose solutions)
		◦ 1,700 mL per exchange
Week 24	9.8	No change
Week 25	12.3	• HD 4 h thrice weekly
		• APD 8 h 4 exchanges (1.36% glucose solutions)
		◦ 1,700 mL per exchange
Week 26	12.3	• HD 6 h 6 times/week
Week 28	6.5	No change
Week 30	7.5	No change
Week 33	9.0	No change
Week 35	6.7	No change
Week 36	6.7	No change

APD = automated peritoneal dialysis; CAPD = continuous ambulatory peritoneal dialysis; HD = hemodialysis.
